# The multisensory basis of the self: From body to identity to others 
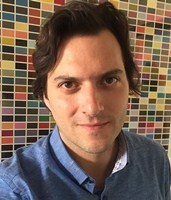


**DOI:** 10.1080/17470218.2016.1181768

**Published:** 2016-05-17

**Authors:** Manos Tsakiris

**Affiliations:** ^a^ Lab of Action & Body, Department of Psychology, Royal Holloway University of London, Egham, Surrey, UK

**Keywords:** Self, Body awareness, Multisensory, Interoception, Social cognition, Body ownership

## Abstract

By grounding the self in the body, experimental psychology has taken the body as the starting point for a science of the self. One fundamental dimension of the bodily self is the sense of body ownership that refers to the special perceptual status of one’s own body, the feeling that “my body” belongs to me. The primary aim of this review article is to highlight recent advances in the study of body ownership and our understanding of the underlying neurocognitive processes in three ways. I first consider how the sense of body ownership has been investigated and elucidated in the context of multisensory integration. Beyond exteroception, recent studies have considered how this exteroceptively driven sense of body ownership can be linked to the other side of embodiment, that of the unobservable, yet felt, interoceptive body, suggesting that these two sides of embodiment interact to provide a unifying bodily self. Lastly, the multisensorial understanding of the self has been shown to have implications for our understanding of social relationships, especially in the context of self–other boundaries. Taken together, these three research strands motivate a unified model of the self inspired by current predictive coding models.

## Experimenting with the self

The self is first and foremost situated within a body. As the leading social psychologist R. Baumeister (1999, p. 2) famously wrote “Everywhere in the world, self starts with body”. Over the last 30 years, several lines of research from what became known as the embodied cognition approach have converged to suggest that cognitive processes are deeply rooted in the body’s interactions with the world (for a review see Wilson, 2002). For example, influential motor theories of perception have inspired new lines of research that allowed us to consider self-awareness and social interactions through the lens of sensorimotor embodied processing (Prinz, 2012). By grounding the self in the body, psychology could, at last, overcome Cartesianism and make the *bodily self* the starting point for a science of the self. One fundamental dimension of the bodily self, but by no means the only one (see, for example, the sense of agency; Synofzik, Vosgerau, & Newen, 2008; Tsakiris, Schütz-Bosbach, & Gallagher, 2007), is the sense of body ownership that refers to the special perceptual status of one’s own body, which makes bodily sensations seem unique to oneself—that is, the feeling that “my body” belongs to me (Gallagher, 2000). The primary aim of this review article is to highlight recent advances in the study of body ownership and our understanding of the underlying neurocognitive processes in three ways. The first step is to consider how the experience of this body as *mine* has been addressed mainly in the context of multisensory integration. The second step is to consider how this exteroceptively driven sense of body ownership can be linked to the other side of embodiment, that of the unobservable, yet felt, interoceptive body. The third step is to consider the implications that this multisensorial understanding of the self has for our understanding of social relationships, especially in the context of self–other boundaries. Taken together, these three research strands motivate the need for the development of a unified model of the self that resonates with current predictive coding models of information processing.

## The exteroceptive model of the bodily selF

The key question of how the brain produces the experience of this body as *mine* has been addressed mainly in the context of multisensory integration. For example, in the rubber hand illusion (RHI; Botvinick & Cohen, 1998), watching a rubber hand being stroked synchronously with one’s own unseen hand causes the rubber hand to be experienced as part of one’s body (for a review see Tsakiris, 2010). Over the last 20 years, the RHI has been established as one of the most important experimental paradigms that allows the controlled manipulation of the experience of body ownership. While the underlying mechanisms and behavioural, physiological, and phenomenological consequences of the illusion have been described in detail elsewhere (Blanke, 2012; Blanke, Slater, & Serino, 2015; Tsakiris, 2010), it is important to highlight here certain key features that are particularly relevant for understanding the relation between the exteroceptive and interoceptive models of the self that will follow in the section “The Interoceptive Model of the Bodily Self”.

First, while not sufficient by itself (see Tsakiris, Carpenter, James, & Fotopoulou, 2010; Tsakiris & Haggard, 2005), synchronous multisensory (i.e., visuotactile in most cases) stimulation is the main cause that drives the RHI and the resulting change in body ownership (Botvinick & Cohen, 1998). Multisensory processing aims at the integration of sensory signals and the resolution of potential conflicts to generate a coherent representation of the world and the body on the basis of the available sensory evidence. The RHI reflects a three-way interaction between vision, touch, and proprioception: Vision of tactile stimulation on the rubber hand captures the tactile sensation on the participant’s own hand, and this visual capture results in a mislocalization of the felt location of one’s own unseen hand towards the spatial location of the visual percept (Botvinick & Cohen, 1998). Second, at the phenomenological level, the RHI has been successfully used as a model instance of embodiment. Longo, Schüür, Kammers, Tsakiris, and Haggard (2008) characterized the subjective experience of body ownership during the RHI, revealing that this consists of distinct components, such as ownership of the limb, its location, and the sense of control over it. Third, the change in body ownership as a result of the RHI can in turn change one’s body image as participants who experienced the RHI perceived their hand and the rubber hand as significantly more similar in terms of their appearance (Longo, Schüür, Kammers, Tsakiris, & Haggard, 2009) than did participants who did not experience the illusion, suggesting that ownership leads to changes in perceived physical similarity.

Beyond these changes, the RHI literature has indicated that the experience of owning the rubber hand results in significant alterations in the way one’s own real hand is processed at the introspective (Longo et al., 2008) and physiological level (Moseley et al., 2008). Introspectively, participants feel as if their own hand had disappeared (Longo et al., 2008), suggesting that the changes caused by RHI do not consist of an addition to or extension of one’s body, but instead they produce incorporation of the foreign hand and replacement of one’s own hand. Intriguingly, the same phenomenon seems to be present at the physiological level. Moseley et al. (2008) provided evidence that the experience of ownership during RHI is also accompanied by significant changes in the homeostatic regulation of the real hand, beyond changes in the subjective experience of one’s body. In particular, skin temperature of the real hand decreased if and when participants experienced the RHI (but see also Kammers, Rose, & Haggard, 2011; Sadibolova & Longo, 2014). Additionally, the magnitude of the decrease in skin temperature on the participant’s own hand was positively correlated with the vividness of the illusion. Importantly, this effect occurred only as a result of the experience of ownership. Thus, a change in conscious experience of body ownership has direct consequences for the homeostatic regulation of real body parts that occur once participants experience the RHI, and not simply as the result of synchronous multisensory stimulation. Even more strikingly, histamine reactivity increased in the “rejected” arm during the rubber hand illusion (Barnsley et al., 2011), implying that the interoceptive system begins to disown the real hand in favour of the prosthetic hand, an effect that recalls Damasio’s definition of “the self” as “whatever the immune system defines as being part of the body” (Damasio, 2003, p. 227).

Therefore, multisensory processes that update or disrupt the awareness of our physical self may in turn disrupt the physiological regulation of the self. The changes caused in the physiological regulation of the self as a result of the change in the conscious experience of body ownership over and above multisensory integration suggest that various processes other than simply multisensory integration may be involved in generating, maintaining, or disrupting the awareness of the bodily self (see the section “The Interoceptive Model of the Bodily Self”).

In terms of the brain areas that underpin body ownership, functional neuroimaging studies on the RHI and lesion studies implicate a network of areas, composed of premotor, temporoparietal, and occipital areas, as well as the insula (for review see Blanke et al., 2015; Tsakiris, 2010). The insula is ubiquitously activated in a wide range of tasks and is typically known as the main interoceptive hub of the brain (for a review see Craig, 2009), but it has been shown that it is also engaged in the experience of body ownership during the RHI (Tsakiris, Hesse, Boy, Haggard, & Fink, 2007). Intriguingly, the hypothesis that the right posterior insula is linked to the experience of body ownership is also supported by available evidence on somatoparaphrenia, a neuropsychological syndrome where loss of experienced ownership over one’s own limb is the key feature. A lesion mapping study that focused on patients with disturbed sense of body ownership (Baier & Karnath, 2008) revealed that the right posterior insula is indeed the critical structure involved in such phenomena.

Beyond ownership of a limb, the same principles of exteroceptive multisensory integration have been used to probe questions of full-body ownership. Ehrsson (2007) used synchronous or asynchronous visuotactile stimulation to elicit out-of-body experiences: Synchronous but not asynchronous visuotactile stimulation induced a shift in the first-person perspective such that participants experienced being located at some distance behind the visual image of their own body as if they were looking at someone else. Lenggenhager, Tadi, Metzinger, and Blanke (2007) induced a full-body illusion (see also Petkova & Ehrsson, 2008) by having participants view a virtual body presented at a distance of 2 m ahead of them through the use a 3D-video head-mounted display. As with the RHI, after synchronous stimulation, participants felt as if the virtual body was their body. Multisensory integration can update the mental representation of one’s body, such as the sense of ownership of body parts (Longo et al., 2008) or whole body (Ehrsson, 2007; Lenggenhager et al., 2007; Petkova & Ehrsson, 2008), the physical appearance of one’s body (Longo et al., 2009), and the more abstract narrative representations of one’s self (Bergouignan, Nyberg, & Ehrsson, 2014). Taken together, these results speak in favour of an exteroceptive model of the self, within which self-awareness is highly malleable, subject to the influence of exteroception (i.e., the perception of the body from the *outside*). However, exteroceptive input represents only one set of channels of information available for self-awareness, as we are also interoceptively aware of our body.

## The interoceptive model of the bodily self

Interoception, as first suggested by Sherrington, who coined the term in 1906 (as cited in Craig, 2009), is the body-to-brain axis of sensations originating from the internal body and its visceral organs that signal their physiological state, such as thirst, dyspnea, “air hunger”, sensual touch, itch, penile stimulation, sexual arousal, coolness, warmth, exercise, heartbeat, distension of the bladder, stomach, and other internal organs. Interoceptive signals arise within four systems—the cardiovascular, respiratory, gastrointestinal, and urogenital. Of those, the cardiovascular has emerged as the main focus of study of the interaction between the visceral body and the brain (Critchley & Harrison, 2013), because of the informationally rich and bidirectional connections between these two most important organs of the body, the heart and the brain. Moreover, psychological research into interoceptive awareness has focused mainly on cardiac awareness because of the known role that heart–brain interactions (and concomitant balance between the sympathetic and parasympathetic systems) play in emotion processing. While vestibular and proprioceptive signals also seem to originate from within, interoception plays a unique role in allowing the brain to ensure the efficient physiological function of the organism (i.e., homeostasis). Interoception is therefore critical for ensuring the *stability* of the organism in a changing environment, in way that other systems are not.

As with awareness of other sensory modalities, awareness of interoceptive states confers significant biological advantages. However, in contrast to the vast empirical data on visual or somatosensory awareness, our understanding of interoceptive awareness is limited by the difficulties in causally manipulating interoceptive states (e.g., controlling inputs to the system), as well as by the available measurement methods. Research into interoceptive awareness has focused mainly on awareness of heartbeats because these are distinct events that can be easily measured, unlike other interoceptive changes. Heartbeat detection procedures typically require individuals to perceive and count the number of heartbeats occurring during short intervals, or to detect the synchronicity or asynchronicity between individual heartbeats and external stimuli. Both methods produce measures of interoceptive accuracy (IAcc), which correlate with each other and with measures in other interoceptive modalities (for review see Garfinkel, Seth, Barrett, Suzuki, & Critchley, 2015). There are significant interindividual differences in performance, allowing us to distinguish between people with higher and lower IAcc. IAcc is thought to reflect a trait-like sensitivity to one’s visceral signals that has important consequences for health, feelings, and cognition. Individual differences in IAcc have been linked to mental health with very high IAcc predisposing to anxiety, while in patients with alexithymia, a condition characterized by difficulties in identifying and describing emotion, symptom severity is inversely related to IAcc. Low IAcc characterizes sufferers from depersonalization disorder, those with personality disorders and psychosomatic complaints, and patients with eating disorders (for a review see Herbert & Pollatos, 2012). In healthy adults, research into interoceptive awareness has been almost exclusively concerned with emotion. IAcc is important for the intensity of emotional experience and emotion regulation (Wiens, 2005). Individuals with higher IAcc report higher arousal than people with lower IAcc, despite comparable physiological arousal, are more able to self-regulate their behaviour (Herbert & Pollatos, 2012), and tend to follow their intuitions more in decision-making tasks (Dunn et al., 2010). Taken together, the available evidence, deriving mainly from the study of emotion and psychopathology, suggests that interoceptive awareness is important for emotional awareness and mental well-being.

However, beyond these domains, cognitive neuroscience has indirectly revealed the ubiquitous role that interoception may play in cognitive processing and self-awareness. Countless functional neuroimaging studies have reported activations in the insula, the central interoceptive hub in the brain, across a wide range of tasks. Craig (2009) suggested that the common denominator of insula activity is the central role of this area in integrating bodily and environmental information to optimize homeostatic efficiency, and in representing the “material me” in the brain. Beyond homeostasis, a set of intriguing findings that relate to self-awareness have captured our attention (for a review see Craig, 2009; Tsakiris, 2010). Right anterior insula activity correlates with performance in IAcc (Critchley, Wiens, Rotshtein, Ohman, & Dolan, 2004). Ronchi et al. (2015) reported a single-case study showing that heartbeat awareness decreased after insular resection. Right mid-posterior insula activity correlates with body ownership experienced during the rubber hand illusion, a paradigm that uses exteroceptive input (e.g., vision and touch) to study the bodily self, and the same area seems to be the critical lesion site for neurological disturbances in the sense of body ownership (Baier & Karnath, 2008). These findings suggest that the interoceptive and the exteroceptive sides of the bodily self are integrated from the posterior to anterior subregions *across* the insular cortex (Simmons et al., 2013), which seems to underpin the experience of this body as *mine*, an experience that is the hallmark of the bodily self. Beyond the representation of the body, the insular cortex is also linked to affective processing of self and others, and a wide range of social cognition processes such as empathy (Bernhardt & Singer, 2012). Taken together, these findings suggest that interoception may play a role for self-awareness that goes beyond its known role for emotion and phenomenal consciousness, and that awareness of the interoceptive body may be fundamental to the *unity* of the self because interoception is required for the experience of a unified, non-hollow self, and to its *stability* because interoception can counteract the ever-changing influence of exteroceptive signals. Notwithstanding the importance of the neuroimaging results showing where in the brain the interoceptive and exteroceptive sides of the self are integrated, they do not answer a fundamental *psychological* question: What is the functional importance of the interactions between the interoceptive and exteroceptive representations *for* the self in its natural embodiment *and* perhaps in the self’s social world?

The first study that tested the potential link between exteroceptive and interoceptive awareness of the body measured and quantified IAcc and compared this measure with the change in body ownership caused by multisensory stimulation, using the RHI as a paradigmatic case of the exteroceptive self. Tsakiris, Tajadura-Jiménez, and Costantini (2011) observed a negative correlation between IAcc and RHI, such that people with lower IAcc showed a stronger RHI measured behaviourally and homeostatically (i.e., drop in skin temperature), suggesting that, in the absence of accurate interoceptive representations, one’s model (i.e., representation) of self is predominantly exteroceptive. This was a seminal finding that it could not be predicted by existing accounts of interoceptive awareness or models of the exteroceptive self. While Moseley et al. (2008) had shown how a change in the body ownership during RHI affects homeostatic regulation, Tsakiris et al. (2011) showed for the first time that both the experience of body ownership and the same subsequent changes in homeostatic regulation are negatively correlated with levels of IAcc. These findings suggested an *antagonism* between interoceptive and exteroceptive cues in bodily self-awareness. Following that initial finding, the same negative correlation was observed in children aged 8 to 17 years (Schauder, Mash, Bryant, & Cascio, 2015).

Since then, recent studies have tried to modify the conditions of multimodal stimulation by including cardiac feedback to examine in greater detail the potential role of interoceptive signals in creating a sense of body ownership. More recent evidence for the integration of exteroceptive and interoceptive information in body ownership comes from two virtual reality studies (Aspell et al., 2013; Suzuki, Garfinkel, Critchley, & Seth, 2013). Suzuki et al. (2013) demonstrated that watching a virtual depiction of their own hand pulsing in synchrony with the participant’s heartbeats induced the subjective experience of ownership over the virtually projected hand. This effect was not observed when the cardiac signals were presented out of synchrony with the participants’ heartbeats. Interestingly, participants with higher IAcc experienced a stronger illusory sense of ownership over the virtual hand than participants with lower IAcc. Contrary to the results of Tsakiris et al. (2011), in this paradigm it was the people with higher IAcc who experienced the greater proprioceptive drift. The important difference between these two experimental manipulations is that in the classic rubber hand illusion the interoceptive cues of the individuals with good heartbeat perception serve to anchor those participants in their own bodies and to enable them to resist the illusion. However, in Suzuki et al.’s novel method, the salient interoceptive cues are now located on the true, filmed hand, predisposing people with higher interceptive awareness to recognize it as their real body part.

Following similar methods of stimulation to those of Suzuki et al. (2013), it was shown that causing an avatar to flash in synchrony with the participant’s own heartbeat (to enhance self-identification with the avatar’s body) facilitates performance on a task that requires participants to judge the perspective of the avatar (Aspell et al., 2013), an effect analogous to the one reported following the classic induction of the full-body illusion (Lenggenhager et al., 2007). In addition to cardiovisual synchrony, respiration can also produce important effects in body awareness. People who saw an image of their own torso flash in synchrony with their respiration experienced a stronger sense of self-location towards the virtual body than when the flashing was asynchronous (and also when compared with an inanimate control object), although they reported no sense of ownership (Adler, Herbelin, Similowski, & Blanke, 2014). Lastly, using another type of interoceptive stimulation—namely, slow affective touch that activates C tactile (CT) afferents—Crucianelli, Metcalf, Fotopoulou, and Jenkinson (2013) and van Stralen et al. (2014) demonstrated how this kind of stimulation enhances the experience of body ownership in the RHI.

These intriguing findings suggest that the integration of sensory information across the interoceptive and exteroceptive domains via cardiovisual synchrony, but also synchrony between respiration and vision, can alter important and diverse facets of body awareness, such as body ownership. Moreover, given that levels of interoceptive accuracy seem to constrain the effects of exteroceptive signals on body awareness, we could argue that, if the exteroceptive model of the self highlights the *malleability* of body awareness given the striking effects that multisensory integration has on body ownership (see the section “The Exteroceptive Model of the Bodily Self”), the interoceptive model of the self seems to primarily serve the *stability* of the body and its mental representation in response to external changes, reflecting thus the biologically necessary balance between adaptability and stability.

## From the bodily self to others: multisensory integration and self–other boundaries

The studies reviewed above have focused mainly on the representation of the self without an explicit reference to others or to social processes. However, self representations are essential not simply for the sake of self-awareness, but more so for the representation of the relationship between self and others, and the effects that such representations have on self–other interactions. A first step towards a more social dimension of the exteroceptive model of the bodily self was to investigate the extent to which current multisensory input may influence self–other boundaries. To that end, beyond ownership over body parts, other studies have used the same method of multisensory integration (i.e., visuotactile stimulation) to ask whether similar changes would occur in the representation of one’s own face. Arguably, one’s face is the body part that most characterizes self-appearance (Rochat, 2015), and recognition of one’s face, as distinctive from others’, is a fundamental component of self-awareness (Gallup, 1970; but see Suddendorf & Butler, 2013). Tsakiris (2008) extended the paradigm of multisensory integration to self-face recognition to investigate whether the process that alters body ownership may also alter more social representations of one’s self. In the enfacement illusion (Apps, Tajadura-Jiménez, Sereno, Blanke, & Tsakiris, 2015; Sforza, Bufalari, Haggard, & Aglioti, 2010; Tsakiris, 2008), watching another person’s face being touched synchronously with one’s own face evokes changes in self-face recognition, so that we perceive the other person’s face as more similar to one’s own. Participants were stroked on their face while they were looking at the face of another unfamiliar individual being touched in synchrony or asynchrony, a procedure that we termed interpersonal multisensory stimulation (IMS). Before and after IMS, participants performed a self-recognition task. The results showed that synchronized multisensory signals had a significant effect on self-face recognition (see also Sforza et al., 2010; Tajadura-Jimenez, Coleman, Longo, & Tsakiris, 2012; Tajadura-Jiménez & Tsakiris, 2014). Not only did participants subjectively rate the other’s face as physically more similar to their own after synchronous IMS (Sforza et al., 2010; Tajadura-Jiménez, Grehl, & Tsakiris, 2012; Tsakiris, 2008), they also showed a shift in their ability to discriminate between their own and the other’s face in a psychophysical visual discrimination task. In one version of this task, participants were shown computer-manipulated images of their own face blended with varying percentages of the other’s facial features using morphing software. Participants were required, for each trial, to report whether the face looked more like their own face, or more like the other’s face. After synchronous IMS, participants accepted a larger percentage of the other’s facial features as their own face (Tajadura-Jiménez, Grehl, et al., 2012), showing an increase in perceived physical self-resemblance of the other. Interestingly, similar to the RHI, individual traits of IAcc were shown to correlate with changes in self–other boundaries during the enfacement illusion (Tajadura-Jimenez, Longo et al., 2012; Tajadura-Jiménez & Tsakiris, 2014), providing further support to the hypothesis that levels of interoceptive awareness may constrain the flexibility of self–other boundaries. Moreover, levels of autonomic arousal (i.e., electrodermal activity) in response to threatening stimuli presented on the model’s face were similarly affected by levels of IAcc; participants with lower IAcc displayed higher arousal to the threat perceived on the other’s face. These effects suggest that the mental representation of our self, such as self-face representation, is not solely derived from stable mnemonic representations, but instead these representations are susceptible to current multisensory evidence.

The changes in perceived physical similarity between self and other in the enfacement illusion was a crucial finding as it suggested that participants’ visual representations of their own and another’s body had become partially overlapped, or shared. In a way analogous to the effects of the RHI, the synchronous IMS in enfacement elicits an overlap, or sharing, of body representations between self and other. Given the putative role of shared body representations in sociocognitive processing (Gallese & Sinigaglia, 2011), the important next step was to investigate how the changes in body representation induced by multisensory integration could affect social cognition.

Recent work has identified a number of social processes that are modulated by synchronous IMS. Early investigations of the experiential structure of the enfacement illusion not only found evidence of changes in perceived physical similarity between self and other, but also revealed a clear affective component, whereby participants perceived the other to be more trustworthy and attractive after synchronous IMS (Tajadura-Jiménez, Longo, Coleman, & Tsakiris, 2012). The social consequences of IMS were further explored in more depth by Paladino, Mazzurega, Pavani, and Schubert (2010) who showed that, following the enfacement illusion, participants rated the other as conceptually closer to themselves, and also attributed to them more self-like personality traits. In addition, Maister, Tsiakkas, and Tsakiris (2013) reported that the illusion can enhance the recognition of emotional facial expressions. After participants experienced the enfacement illusion with an unfamiliar individual, they showed significant increases in speed and accuracy in the recognition of the facial expressions of that individual, specifically their expressions of fear.

A more direct approach to social processes was proposed by Maister, Sebanz, Knoblich, and Tsakiris (2013) who asked whether changes brought about by multisensory integration can extend to the social processing of an entire group. Light-skinned Caucasian participants experienced the rubber hand illusion over a dark-skinned hand, and the change in their implicit racial attitudes was measured using the Implicit Association Test (IAT). The experience of illusory ownership over the different-race hand was strongly correlated with increased implicit positive attitudes towards that race. Similar findings were subsequently reported using a virtual reality set-up in which participants embodied a different-race avatar. Again, changes in body ownership elicited by the procedure led to a decrease in implicit racial biases against the embodied racial group (Peck, Seinfeld, Aglioti, & Slater, 2013), and similar effects were more recently reported for age-stereotypes (Banakou, Groten, & Slater, 2013) and other higher order social and attitudinal processes (Osimo, Pizarro, Spanlang, & Slater, 2015). For example, embodying an avatar representing a 4-year-old child resulted in a bias towards associating the self with child-like compared to adult-like categorizations, as measured using an IAT (Banakou et al., 2013). This study was notable because it demonstrated a role of the self-association in attitude change, whereas previous research had investigated more generic positive or negative associations with the embodied social group.

Taken together, these studies demonstrate that bodily illusions not only alter higher level representations of the self and the other such as social or racial stereotypes, but also more fundamental, low-level processes in perceived physical similarity, online social perception, and attention. Although IMS affects a wide range of diverse social processes, these effects can be parsimoniously explained by an increase in perceived physical self-resemblance, as it has been shown that such changes in body ownership or self-face representations that allow us to incorporate another body or face may also increase “bodily resonance” with that outgroup. Our perceptual and neural resonance with others’ bodily experiences is significantly reduced when observing an outgroup member (Avenanti, Sirigu, & Aglioti, 2010; Gutsell & Inzlicht, 2010; Serino, Giovagnoli, & Làdavas, 2009; Xu, Zuo, Wang, & Han, 2009). An example of this can be seen in the visual remapping of touch effect, a phenomenon whereby our tactile sensitivity is enhanced when observing another person being touched. This effect, thought to be evidence of somatosensory resonance with others, is significantly reduced when the observed individual is a member of a racial or political outgroup (Serino et al., 2009). In a recent study, the enfacement illusion was induced by exposing participants to two minutes of multisensory stimulation whilst viewing an out-group member’s face (Fini, Cardini, Tajadura-Jimenez, Serino, & Tsakiris, 2013; see also Cardini, Tajadura-Jiménez, Serino, & Tsakiris, 2013). Immediately afterwards, participants’ tactile sensitivity was measured whilst they observed the out-group member’s face being touched. Results showed that the experience of identification with the out-group member’s face had increased the visual remapping of touch effect up to the level normally associated with a same-race individual.

These findings show that a change in the perception of a purely bodily aspect of the self, such as the ownership of one’s hand or the mental representation of one’s face, ultimately alters not only associations with a higher level concept of the self (Banakou et al., 2013), but also the affective (Maister, Tsiakkas, et al., 2013) and social processing of others (Maister, Tsiakkas, et al., 2013). What is the underlying mechanism that allows changes in body ownership and self-representation to influence the way we perceive and relate to others?

As discussed earlier, experimentally induced modulations of body ownership and self-face representations enhance perceived physical similarity between self and other (Longo et al., 2009; Sforza et al., 2010; Tajadura-Jiménez, Grehl, et al., 2012; Tsakiris, 2008). It has been argued that such changes in perceived physical similarity between self and other can in turn lead to new higher level positive associations being formed between the self and others (Maister, Slater, Sanchez-Vives, & Tsakiris, 2015). The transition from bodily to more conceptual representations of self and other can be explained by the fact that others who come to be perceived as more physically similar to one’s self may activate self-representations. Even subliminal exposure to images of one’s own body automatically activates positive self-concepts (Ma & Han, 2010; Tao, Zhang, Li, & Geng, 2012), and therefore perceptions of self-similar bodies may activate self-associations in the same way. Once self-concepts have been activated by others, the positive evaluations associated with self-concepts can be generalized to others, by virtue of their perceptual similarity to the self. In support of this, the classical conditioning literature has long posited that associative learning of likes and dislikes is based on perceptual similarity, and that this can occur outside of awareness (for a review see De Houwer, Thomas, & Baeyens, 2001). This process of evaluative conditioning has been shown to extend to social stimuli; individuals rapidly and unintentionally generalize affective processing to individuals who look physically similar (Verosky & Todorov, 2010). Such processes can explain how a newly established physical similarity between self and others can lead to a change in the conceptual representations of self and others. In addition to the reported social effects, the process described above points to the need to account for a plausible mechanism that can underpin the multifaceted aspects of one’s self and its body, from higher level (e.g., identity, stereotypes) to lower level (e.g., appearance) mental representations. Such accounts have been recently proposed within the framework of predictive coding.

## Towards a unifying theory of the (bodily) self

The idea that there is more than one side to the self is as old as psychology but theories that integrate the interoceptive and exteroceptive sides of self have been lacking (Legrand & Ruby, 2009). Some theories have focused mostly on the idea of coherence (Zahavi, 2005), thus neglecting the fact that certain aspects of the self may be antagonistic or competitive. Others (Metzinger, 2003), inspired by Hume, have focused on antagonism to argue against the unity of the self. We here provide a different approach by focusing on the *dynamic* relations between bodily modalities, so that processes of both *integration* and *competition* can be accounted for, contributing to the *unity* and *stability* of the bodily self, respectively.

Predictive coding (PC) has emerged as a prominent unifying theory of cortical function to explain brain processes underlying perception, action, and (more recently) interoception (Friston, 2010; Seth, 2013). According to the theory, incoming sensory data are compared with internal models—that is, with the brain’s probabilistic “prediction” (best guess) about the environmental causes that affect the organism’s nervous system. If predictions and data are not compatible, then “prediction errors” arise. However, organisms must maintain their bodies within a narrow range of desirable states, and therefore prediction errors must be minimized. A central tenet of PC models is the free-energy principle that states that biological agents resist a natural tendency towards disorder in a constantly changing environment (Friston, 2005), which may also have considerable consequences for the stability of self-representations. In the long term, this means that the brain as a whole minimizes the average of surprise across all sensory systems, learning how best to model and predict incoming sensory input.

We recently extended this framework to self-awareness to account for the malleability of the exteroceptive self (Apps & Tsakiris, 2014) and argued that one’s body is processed in a probabilistic manner as the most likely to be “me” (see Limanowski & Blankenburg, 2015, for empirical support). Such probabilistic representations are created through the integration of top-down “predictions” about the body with bottom-up “prediction errors” from unimodal sensory systems that are then explained away at higher levels of the hierarchy (i.e., in multisensory areas). In the case of bodily illusions, viewing touch on a different body evokes a sensation of touch on one’s own body, and this generates bottom-up error signals from unimodal sensory systems. Perceptual learning processes will update the body representation to first induce a sense of ownership over the new body and next to incorporate perceptual features of the other’s body, in order to minimize this error and maintain a continual sense of “mineness”. Therefore, this account can explain how synchronous multisensory stimulation, such as the one provided during the bodily illusions, can not only elicit fundamental changes in body ownership, but can also elicit a subsequent increase in perceived similarity between the bodies of self and other.

Of course, one’s self is not represented solely at a basic, perceptual level. The self is a multimodal, hierarchical construct containing both low-level, bodily representations as well as higher level attitudes and beliefs. On a predictive coding account, these different levels of representation continuously interact, as prediction errors, and when left unexplained at one level, they need to be processed and eliminated at a higher level of the hierarchy. Given the focus of PC accounts on complementary hierarchical top-down and bottom-up processes, a change in low-level, perceptual representations of one’s own body in relation to the body of other creates errors further up in the processing hierarchy, as this new information now conflicts with more abstract, higher order representations of oneself and the other. These errors must then be minimized in a similar way, by updating attitudes and beliefs held about one’s self and the other, ensuring that the consistency within the multimodal self-representation is maintained (see also Moutoussis, Fearon, El-Deredy, Dolan, & Friston, 2014, for a predictive coding approach to social cognition).

Beyond the explanatory value that predictive models of the self have for the malleability of body awareness and self–other representations, such models can also be used to account for the relation between exteroceptive and interoceptive representations of one’s body. For example, if we consider the experience of body ownership during the RHI, the exteroceptive evidence suggests that what I am looking at (i.e., the rubber hand) is my hand. However, if this is my hand, then there are interoceptive prediction errors that need to be explained away between how my true hand feels (i.e., the interoceptive prediction) and the fact that I cannot feel the rubber hand interoceptively (i.e., the interoceptive prediction errors). An important contribution of such free energy models (see also Seth, 2013) is the proposal that the self is hierarchically distributed and underpinned by many different types of information. Signals and predictions from any modality may thus be brought to bear to resolve a conflict between cues in another, including higher level, abstract, and amodal assumptions (predictions). Therefore, exteroceptive and interoceptive streams must be integrated for a body to be represented as “self”. What determines the weighting of the different streams?

Both predictions and the incoming sensory data vary in the precision (i.e., reliability) of the information that they convey (e.g., how noisy they are). Sensory signals (and consequent prediction errors) that are compatible with only a narrow range of potential predictions have “high precision” and thus carry information that is reliable. By contrast, sensory signals with “low precision” are compatible with a wide range of potential predictions such that the resulting imprecise prediction errors they set up are likely to be treated as unreliable information and are consequently suppressed by a precise prediction. Precision is crucial when selecting information amongst various modalities because the brain preferentially weights signals that are the most precise in the given context. To experience body ownership during the RHI, participants must form a percept that the prosthetic hand is their own, by minimizing prediction errors across all available sensory modalities. Importantly in this context, information from any sensory modality can be used to explain away prediction error in any other (Apps & Tsakiris, 2014). It is precision that dictates which part of the conflicting evidence is presumed to be reliable. If their interoceptive signals are precise, this would explain why individuals with high IAcc experience a weaker body ownership during the RHI by contrast with individuals with low IAcc. For higher IAcc the brain weights interoceptive predictions and prediction errors as more reliable than it does for those with low IAcc, making them more resistant to the exteroceptive evidence. This account can also explain why in the case of “interoceptive rubber hand illusion” (Suzuki et al., 2013; see also Aspell et al., 2013, for a similar full-body illusion) it is the people with higher interoceptive accuracy who now experience the greater illusion, illustrating the crucial effect of context. The interoceptive cues in this version of the experiment indicate that the hand is one’s own because its visual appearance is congruent with the individual’s continually experienced updating of interoceptive priors. People for whom interoceptive prediction errors are precise (i.e., good heartbeat perceivers) are therefore now more, rather than less, likely to claim ownership of the virtual hand, as this experiment has demonstrated (Suzuki et al., 2013). Therefore, individual differences in IAcc accuracy can be explained in terms of variations in the “precision” with which interoceptive signals from within the body are represented (Fotopoulou, 2013; Friston, 2010; Seth, 2013), and this precision-dependent account can also explain the effects that levels of IAcc may have on the exteroceptively driven representation of the self.

Beyond the self, the distinction between self and other is crucial both for self-awareness and for awareness of other people because the brain must monitor whether sensations, events, and mental states should be attributed to oneself or not. Correctly identifying the origin of bodily and mental states is necessary for social relatedness. For example, how can I share the pain of another individual without forgetting it is not my pain? Emotional contagion, mimicry, body resonance, perspective taking, and theory of mind have been used to operationalize different facets of empathy, which is considered as one of the hallmarks of social relatedness (Bernhardt & Singer, 2012; Decety, 2011), although its exact meaning is still debated. Aside from definitional disputes, a critical but unresolved issue is the question of “self–other” overlap (Preston & Hofelich, 2012). Put simply, “self–other” overlap is thought to arise when an observer engages in an isomorphic state (e.g., same emotion) to the person observed. However, what is or should be the extent of this overlap? Or, to turn the question around, to what degree can we distinguish between self and other at the very time that we are trying to relate to each other?

The model put forward here proposes a role for interoception as a constituting element of the stability of bodily self that safeguards against self–other blurring. This paves the way for a new, hitherto untested, approach to the question of self–other relations. According to recent models of social cognition (Bird & Viding, 2014), the default modus operandi of the social brain is to represent one’s own self (e.g., one’s own perspective, emotion, beliefs). Switching from self to other, to achieve a partial co-representation of self and other, is therefore an effortful process that at least to some extent requires the attenuation of self-representations (Bird & Viding, 2014). From the interoceptive viewpoint, the attenuation of self-representations that is required so that the other is better represented needs to be extended to interoceptive feelings. According to this view, low IAcc may provide an advantage because the attenuation of interoceptive prediction errors may be computationally easier. Consider the case of emotion contagion, where exposure to someone else’s emotion brings about a similar affective state in the perceiver but without explicit awareness that this state should be attributed to the other individual. A lack of awareness of the origin of the affective state may indicate low IAcc, despite comparable levels of physiological reactivity between people with lower and higher IAcc (Dunn et al., 2010). In the case of empathy, which, unlike emotion contagion, requires explicit knowledge of the origin of the emotion, it is unclear, and as yet empirically untested, whether one needs first and foremost an accurate sense of one’s own body, as it is being affected by others, in order to sense the other.

An alternative prediction, motivated by the way in which interoception is conceptualized here, holds that understanding others requires a “good enough” (i.e., precise) representation of one’s own (interoceptive) states because the key element in representing others’ states is our awareness of how *their* states affect *us*. Moreover, this self-representation should display sufficient stability to prevent the blurring of self and other. Therefore, the hypothesis supported by the conceptualization of interoceptive awareness offered here is that one needs an accurate sense of one’s own body, in order to attend to and relate to others, as individuals distinct from one’s self. Therefore, future studies on interoceptive awareness in social cognition can inform a new model of social relatedness motivated by the role that interoceptive representations play in shaping our sense of self and, as a consequence, the ways in which the self relates to others.

## Conclusion

The predictive coding account of self-awareness presented here provides a plausible explanation of the often striking effects that have been reported in relation to bodily illusions over the last 20 years, as it explains how exteroceptive evidence can be used to minimize prediction errors during the construction of our body awareness but also how exteroceptive information is integrated with interoceptive information in this process. In order to minimize prediction errors, the organism must learn over time to assign the best possible set of the weights and thus to optimize the relative precisions of predictions and prediction errors across all modalities. This process has large explanatory value when considering the interaction between interoceptive stability and the malleability of the exteroceptive self, as it explains individual differences in interoceptive or exteroceptive precision and their effects for the awareness of the bodily self, and of others. By appealing to a multidimensional self-representation, in which both bodily and more abstract aspects of the self are bound in a coherent, supramodal construct, we can bridge the gap between multisensory (i.e., interoceptive and exteroceptive) representations involved in body ownership on the one hand, and the more conceptual or social representations involved in self–other relations on the other hand. The mechanisms suggested here provide us with an account of how changes in body ownership can close this gap in order to affect higher level social processes. However, important questions remain unanswered. Presumably, trait levels of interoceptive awareness depend on specific developmental trajectories that till hitherto remain largely underinvestigated. Furthermore, despite the general appeal that predictive coding models have for researchers in the field of body- and self-awareness, direct empirical support has only recently started to be generated. Such challenges should and will be addressed in the near future.
